# *C9orf72* hexanucleotide repeat allele tagging SNPs: Associations with ALS risk and longevity

**DOI:** 10.3389/fgene.2023.1087098

**Published:** 2023-03-01

**Authors:** Karri Kaivola, Matti Pirinen, Hannu Laaksovirta, Lilja Jansson, Osma Rautila, Jyrki Launes, Laura Hokkanen, Jari Lahti, Johan G. Eriksson, Timo E. Strandberg, FinnGen, Pentti J. Tienari

**Affiliations:** ^1^ Translational Immunology, Research Programs Unit, University of Helsinki, Helsinki, Finland; ^2^ Department of Neurology, Helsinki University Hospital, Helsinki, Finland; ^3^ Institute for Molecular Medicine Finland (FIMM), Helsinki Institute of Life Science (HiLIFE), University of Helsinki, Helsinki, Finland; ^4^ Department of Public Health, University of Helsinki, Helsinki, Finland; ^5^ Department of Mathematics and Statistics, University of Helsinki, Helsinki, Finland; ^6^ Department of Psychology and Logopedics, University of Helsinki, Helsinki, Finland; ^7^ Folkhälsan Research Center, Helsinki, Finland; ^8^ Singapore Institute for Clinical Sciences, Agency for Science Technology and Research, Singapore, Singapore; ^9^ Department of Obstetrics and Gynaecology, Yong Loo Lin School of Medicine, National University of Singapore, Singapore, Singapore; ^10^ Department of General Practice and Primary Healthcare, University of Helsinki, Helsinki, Finland; ^11^ University of Helsinki and Helsinki University Hospital, Helsinki, Finland; ^12^ University of Oulu, Center for Life Course Health Research, Oulu, Finland

**Keywords:** *C9orf72*, ALS, intermediate allele, survival, case-control analysis, biobank

## Abstract

*C9orf72* hexanucleotide repeat expansion is a common cause of amyotrophic lateral sclerosis (ALS) and frontotemporal dementia (FTD). The *C9orf72* locus may harbor residual risk outside the hexanucleotide repeat expansion, but the evidence is conflicting. Here, we first compared 683 unrelated amyotrophic lateral sclerosis cases and 3,196 controls with Finnish ancestry to find best single nucleotide polymorphisms that tag the *C9orf72* hexanucleotide repeat expansion and intermediate-length alleles. Rs2814707 was the best tagging single nucleotide polymorphisms for intermediate-length alleles with ≥7 repeats (*p* = 5 × 10^−307^) and rs139185008 for the hexanucleotide repeat expansion (*p* = 7 × 10^−114^) as well as alleles with ≥20 repeats. rs139185008*C associated with amyotrophic lateral sclerosis after removing cases with the hexanucleotide repeat expansion, especially in the subpopulation homozygous for the rs2814707*T (*p* = 0.0002, OR = 5.06), which supports the concept of residual amyotrophic lateral sclerosis risk at the *C9orf72* haplotypes other than the hexanucleotide repeat expansion. We then leveraged Finnish biobank data to test the effects of rs2814707*T and rs139185008*C on longevity after removing individuals with amyotrophic lateral sclerosis / frontotemporal dementia diagnoses. In the discovery cohort (*n* = 230,006), the frequency of rs139185008*C heterozygotes decreased significantly with age in the comparisons between 50 and 80 years vs. >80 years (*p* = 0.0005) and <50 years vs. >80 years (*p* = 0.0001). The findings were similar but less significant in a smaller replication cohort (2-sided *p* = 0.037 in 50–80 years vs. >80 years and 0.061 in <50 years vs. >80 years). Analysis of the allele frequencies in 5-year bins demonstrated that the decrease of rs139185008*C started after the age of 70 years. The hexanucleotide repeat expansion tagging single nucleotide polymorphisms decreasing frequency with age suggests its’ association with age-related diseases probably also outside amyotrophic lateral sclerosis / frontotemporal dementia.

## Introduction

The *C9orf72* hexanucleotide repeat expansion (HRE) is the most common genetic cause of amyotrophic lateral sclerosis (ALS) and frontotemporal dementia (FTD) in populations of European descent (Renton et al., 2011; Majounie et al., 2012) and especially common in Finland (Laaksovirta et al., 2022). The same mutation has also been reported, although less commonly, in other neurodegenerative conditions, such as Alzheimer’s disease, parkinsonism, Huntington-like, corticobasal syndrome, olivopontocerebellar degeneration and idiopathic normal pressure hydrocephalus (Beck et al., 2013; Liu et al., 2014; Korhonen et al., 2019; Kohli et al., 2013; Lindquist et al., 2013; O'Dowd et al., 2012; Xi et al., 2012).

The hexanucleotide repeat alleles can be broadly categorized into small (2-6 repeats), intermediate-length and expansion alleles. The exact threshold of an expansion has not been fully defined but the expansion usually consists of hundreds or thousands of repeats and exhibits somatic mosaicism (Beck et al., 2013).

In addition to the HRE, intermediate-length alleles have also been associated with various diseases, although inconsistently. These include both neurodegenerative (Ng and Tan, 2017) and immunological diseases (Fredi et al., 2019). Immunological disease could potentially develop by alterations in the expression of *C9orf72*, it has been shown that mice with *C9orf72* knockdown develop a fatal autoimmune disease (Atanasio et al., 2016; Burberry et al., 2016). The intermediate-length alleles often occur on the same haplotype as the HRE and there is evidence that DNA methylation and gene expression differs in the intermediate-length alleles as compared to small alleles (Gijselinck et al., 2016).

We have recently reported that in the Finnish population carriership of two intermediate-length alleles is a risk factor for ALS, especially when one of the alleles is ≥ 17 repeats. Similarly, we observed an increased risk for ALS [odds ratio (OR) 1.89, *p* = 0.018] in individuals homozygous for the intermediate allele tagging single-nucleotide polymorphism (SNP) rs3849942 after excluding carriers of the HRE (Kaivola et al., 2020). Similar findings have been previously reported in other populations, too. Van der Zee et al. (van der Zee et al., 2013) reported that homozygosity for a SNP (rs2814707) was associated with FTD in a Flanders-Belgian case-control study (OR 1.75, *p* = 0.04) after excluding expansion carriers. In Belgian ALS and FTD–ALS patients a significantly increased risk was found for carriers of two copies of the intermediate length alleles (OR 2.08, *p* = 0.04) (Gijselinck et al., 2016). These findings suggest that there may be residual risk for ALS/FTD at the *C9orf72* locus, other than the HRE. This residual risk could play a role in other diseases, too.

Here, we have first analyzed best tagging SNPs for the *C9orf72* intermediate-length alleles and HRE in Finnish ALS cases and controls. Then, we studied if the allele frequencies of these SNPs decrease with age in a large biobank dataset from Finland (FinnGen) after removing individuals with the diagnosis of ALS or FTD to observe possible effect on longevity outside the ALS-FTD spectrum.

## Method

### Study cohorts

#### ALS case-control cohort and genotyping

To identify the best tagging SNPs for *C9orf72* intermediate-length alleles and expansion, we used previously published cohorts (Kaivola et al., 2020) of 705 unrelated ALS with Finnish ancestry and 3,196 controls with genotype data available. All *C9orf72* hexanucleotide repeat allele length assessments were done in the same laboratory. Repeat-primed PCR (RP-PCR) was used and all samples with putative alleles of ≥20 repeats including HRE were tested with over-the-repeat PCR. Samples that showed the typical saw tooth pattern in RP-PCR and did not produce longer amplicon in over-the-repeat PCR were categorized as expansions. The longest non-expanded (amplifiable) discrete allele we could detect in controls was 45 repeats, and we used it as the expansion threshold (Kaivola et al., 2019).

Genome-wide genotyping was performed according to manufacturer’s instructions. All controls were genotyped with Illumina genotyping arrays (three cohorts with Illumina Global Screening Array 24v2-3, one with Illumina HumanCNV370 array, one with Illumina 610 k array) and ALS cases were genotyped with Affymetrix Axiom custom SNP array. Samples genotyped with the same genotyping array were processed together. Genotyping data underwent standard per-sample and per-variant quality control steps (Supplementary Material) (Anderson et al., 2010). To analyze SNPs that were not covered by the genotyping arrays, we imputed SNPs using a Finnish reference panel (dx.doi.org/10.17504/protocols.io.xbgfijw). After imputation, in each batch we included variants with a minor allele count >3 and imputation INFO score≥0.90. Then, all batches were merged and variants with an overall genotyping rate >0.95 that were within +- 6 Mb of the *C9orf72* risk haplotype (chr9:21547063-33546474, hg38) were included in subsequent analyses. Additionally, two SNPs (rs147211831 and rs117204439) identified in a previous European study to associate with FTD and intermediate allele length (Reus et al., 2021) were included in the study albeit their imputation INFO scores were not≥0.90 in all cohorts (≥0.70 in all cohorts). These SNPs were included to test possible population differences in the haplotype backgrounds.

#### Biobank cohorts

To test the effect of *C9orf72* intermediate-length alleles and HRE tagging SNPs on longevity, we used FinnGen (https://www.finngen.fi/en) (Kurki and Palta, 2022) data for building discovery and replication cohorts. Samples in FinnGen originate from prospective epidemiological cohorts, disease-based cohorts, and hospital biobank samples. In FinnGen, imputed genotype data is integrated with data from national registries such as hospital discharge records, cause of death registry and medicine reimbursement registry.

The discovery cohort was built using samples from FinnGen release 9. To reduce possible bias from population stratification and relatedness, we excluded related samples and samples without Finnish ancestry (*n* = 144,031). Then, we excluded samples with a diagnosis of motor neuron disease/Duchenne muscular dystrophy (ICD-10 codes G12.2 or G71.06, ICD-9 code 3352A or ICD-8 code 348[0-1]34821) or frontotemporal dementia (wide definition, ICD-10 codes F02.0 or F02.9, ICD-9 code 3311, ICD-8 code 29011) (*n* = 369). After exclusions, our discovery cohort consisted of 232,878 samples.

For replication cohort, we used FinnGen release 10 data and selected 258,910 unrelated individuals with Finnish ancestry. Then, we excluded individuals with ALS and FTD diagnosis (*n* = 413). Finally, we excluded individuals analyzed in release 9 data (*n* = 177,455) leaving 81,042 individuals.

#### Statistical analyses

In our ALS case-control cohort, we used R v. 4.2.1 (RCT, 2022) and PLINK2 (Chang et al., 2015) to perform logistic regression analyses on ALS patients vs. controls and intermediate-length allele (7-45 repeats) carriers vs. non-carriers after exclusion of expansion carriers. We also tested ALS cases with expansion *versus* controls without expansion. Since we wanted to test only the association between genetic variants and *C9orf72* allele length, we did not include covariates in our regression model.

In FinnGen biobank data, to study if rs139185008*C and rs2814707*T heterozygosity or rs2814707*T allele homozygosity affect longevity, we binned samples into three age groups: under 50 years, 50–80 years and over 80 years. The age thresholds were based on age quartiles (first quartile 48 years, third quartile 74 years) and on the rationale that ALS and FTD are relatively rare under the age of 50 years but almost all are diagnosed by 80 years (Chang et al., 2015). In Finland the age-of-onset of ALS is under the age of 50 in ca. 20% of carriers of the C9orf72 HRE (Laaksovirta et al., 2022). We also performed an additional analysis across all ages in which we divided individuals into five-year bins between 20 and 95 years. We excluded bins <20 years and >95 years since they were small (*n* < 1000). We then estimated the allele frequencies with 95% confidence intervals using binom.test function in R in the age bins. We then fitted a logistic regression model that explained the minor allele status (1/0) by the age of the corresponding individual and reported the *p*-value of the age effect in the discovery (N = 230,006) and replication cohort (N = 80,012). Age was defined as the age-of-death or age at the end of follow-up. In discovery cohort, we performed six independent tests and set the threshold for statistical significance to 0.05/6 = 0.0083. In replication cohort, the threshold for statistical significance was 0.05.

#### Ethics

The ALS case-control study was approved by the Ethics Committee of the Helsinki University Hospital (diary number 401/13/03/01/09 and HUS/1720/2019). All individuals or their next-of-kin gave a written informed consent.

The ethics declarations for FinnGen biobank data are provided in Supplementary Material.

## Results

We used two different cohorts, a Finnish ALS case-control cohort and a Finnish biobank cohort. The purpose of the case-control analysis was to 1) identify best SNPs tagging the C9orf72 HRE and intermediate-length alleles, 2) two analyze the association of these SNPs with ALS risk after exclusion of cases with HRE and 3) to analyze the association with ALS risk in Finland using the top SNPs identified in other European populations.

The Finnish biobank data was used to analyze the association of the tagging SNPs with age by comparing their frequencies in different age groups.

### ALS case-control cohort: *C9orf72* hexanucleotide repeat allele tagging SNPs

**FIGURE 1:**
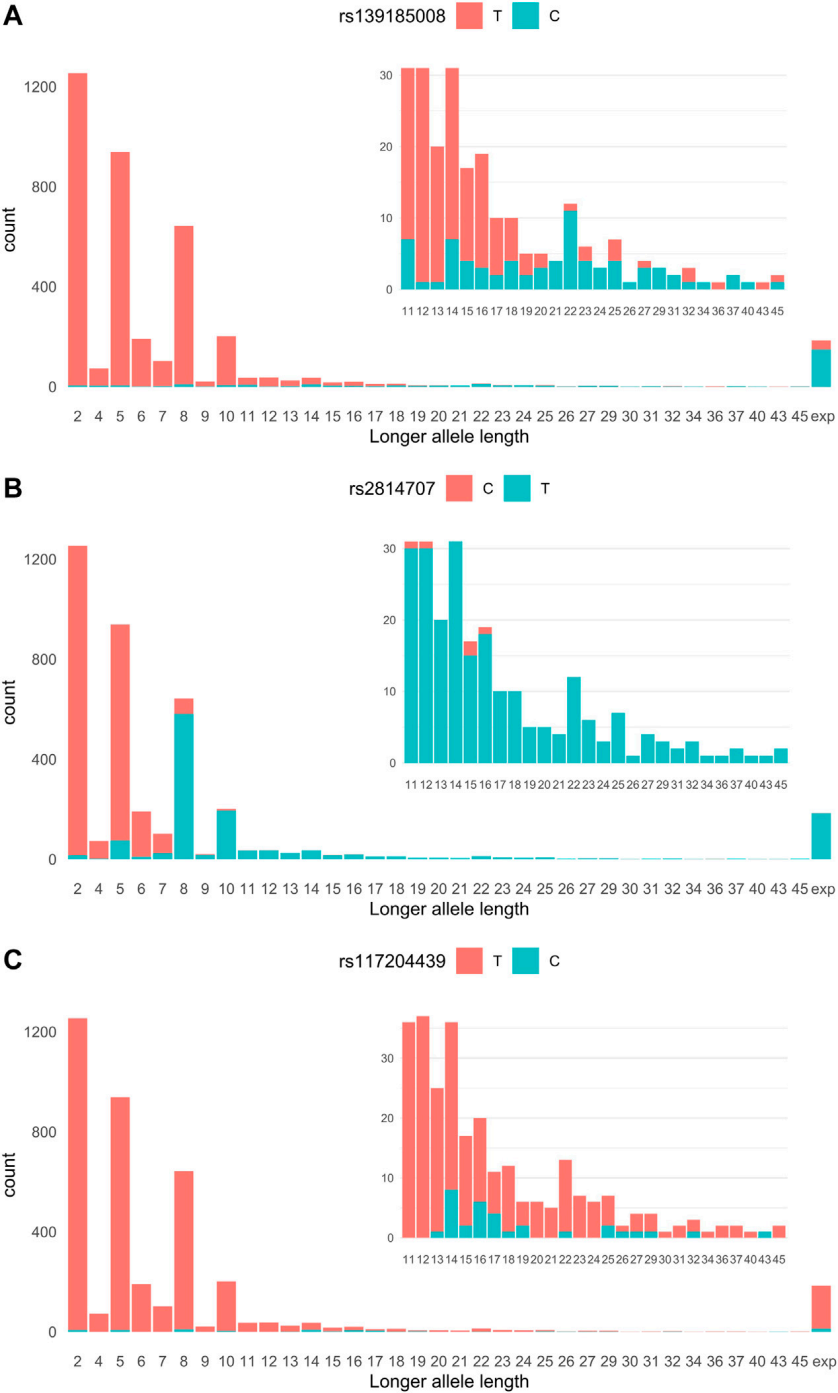
**(A)** rs2814707 **(B)** rs139185008 and **(C)** rs117204439 associations with *C9orf72* hexanucleotide repeat allele length. Statistics for the association with intermediate-length alleles: rs147211831 *p* = 2.47 × 10^−4^, OR = 3.45, 95% CI 1.78–6.70, MAF 1.0% in intermediate allele carriers vs. 0.28% carriers of 2-6 repeats, and rs117204439 *p* = 9.38 × 10^−10^, OR = 6.59, 95% CI 3.60–12.05, MAF 1.0% in intermediate allele carriers vs. 0.28% carriers of 2-6 repeats, and rs117204439 *p* = 9.38 × 10^−10^, OR = 6.59, 95% CI 3.60–12.05, MAF 1.82% in intermediate allele carriers vs. 0.028% in carriers of 2-6 repeats. Statistics for the association with the HRE: rs147211831 *p* = 1.32 × 10^−7^, OR = 6.67, 95% CI 3.30–13.5, MAF 3.1% in expansion carriers vs. 0.49% in non-expansion carriers and rs117204439 *p* = 1.44 × 10^−6^, OR = 4.59, 95% CI 2.47–8.54, MAF 3.6% in expansions carriers vs. 0.78% in non-expansion carriers. Exp: expansion.

We imputed 24,089 genotypes at the *C9orf72* locus of 683 ALS cases and 3,196 controls whose *C9orf72* hexanucleotide repeat alleles we had previously determined (Kaivola et al., 2020). The most significant association with ALS was found with rs139185008 [*p* = 6.45 × 10^−69^, OR = 10.84 95% confidence interval (CI) 8.31–14.15]. Figure 1A shows the relationship of rs139185008 alleles with intermediate-length alleles according to longer allele length. Out of the 179 ALS patients with the HRE, 80% carried rs139185008*C (minor allele), which also tagged longer intermediate-length alleles, especially those with ≥20 repeats (Figure 1A). When testing separately 179 ALS expansion carriers *versus* 3,190 non-carriers, rs139185008 was expectedly the leading expansion tagging variant (*p* = 6.70 × 10^−114^, odds ratio (OR) = 136.34, 95% confidence interval (CI) 89.19–208.57).

Rs139185008 has been previously reported as the top HRE tagging SNP in Finland and associated with idiopathic normal pressure hydrocephalus as well as with FTD (3 × 10^−15^, OR 4.38) and ALS (3 × 10^−21^, OR 5.19) in the FinnGen release 5 (Korhonen et al., 2019; Rostalski et al., 2021).

We next analyzed SNPs that associate with carriership of intermediate-length alleles (carriers of the HRE were excluded). We compared SNPs in carriers of 7–45 repeat alleles (*n* = 1,237) vs. non-carriers (*n* = 2,457) and identified rs2814707*T as the leading intermediate-length allele tagging variant (*p* = 5.44 × 10^−307^, OR = 130.76, 95% CI = 101.79–169.57). Rs2814707*T was found in 87% of the 7-45 repeat allele carriers and 93% of 8–45 repeat allele carriers (Figure 1B). As seen in Figure 1B this marker is mainly tagging alleles with ≥8 repeats and is present in 100% of the HRE carriers.

### ALS case-control cohort: *C9orf72* locus association with ALS after exclusion of carriers of *C9orf72* HRE

We have previously reported in a largely overlapping data set that two copies of the *C9orf72* intermediate-length alleles, especially when the longer allele is ≥ 17 repeats—and homozygosity for the minor allele of rs3849942 (in LD with rs2814707)—associate with ALS risk after exclusion of HRE carriers (Kaivola et al., 2020). Here we extend these finding by analyzing rs2814707 and rs139185008 in non-carriers of the HRE to validate our previous observations based on direct *C9orf72* repeat length assessments and explore putative haplotype effects.

The carrier frequency of rs2814707*T was 35.7% (180/504) in ALS cases and 31.4% (1002/3190) in controls (*p* = 0.057, OR = 1.21, 95% CI 0.99–1.48). Rs2814707*T homozygosity was found in 5.0% (25/504) of ALS cases and in 2.8% (88/3190) of the controls (*p* = 0.012, OR = 1.84, 95% CI 1.17–2.90). These results are virtually the same as we have reported before for rs3849942 (Kaivola et al., 2020).

The carrier frequency of rs139185008*C was 6.0% (30/504) in ALS cases and 2.9% (91/3190) in controls (*p* = 0.0010, OR = 2.15, 95% CI 1.36–3.33). Rs2814707*T homozygosity in combination with rs139185008*C carriership was more common in ALS than in controls (*p* = 0.00020, OR = 5.06, 95% CI 2.06–12.07). In contrast, there was no statistically significant difference in the frequency of rs2814707*T homozygotes after removing rs139185008*C carriers between ALS cases (2.8%, 14/504) and controls (2.3%, 74/3190) (*p* = 0.53, OR = 1.20, 95% CI 0.62–2.17). As rs139185008*C tags especially the longer alleles these results indicate that a genotype with two copies of intermediate-length alleles is a risk factor for ALS, when at least one longer allele is present. These SNP-based results are consistent with our previous results obtained by intermediate-length allele genotyping (Kaivola et al., 2020).

### ALS case-control cohort: Comparative analysis of tagging SNPs discovered in other populations

**FIGURE 2:**
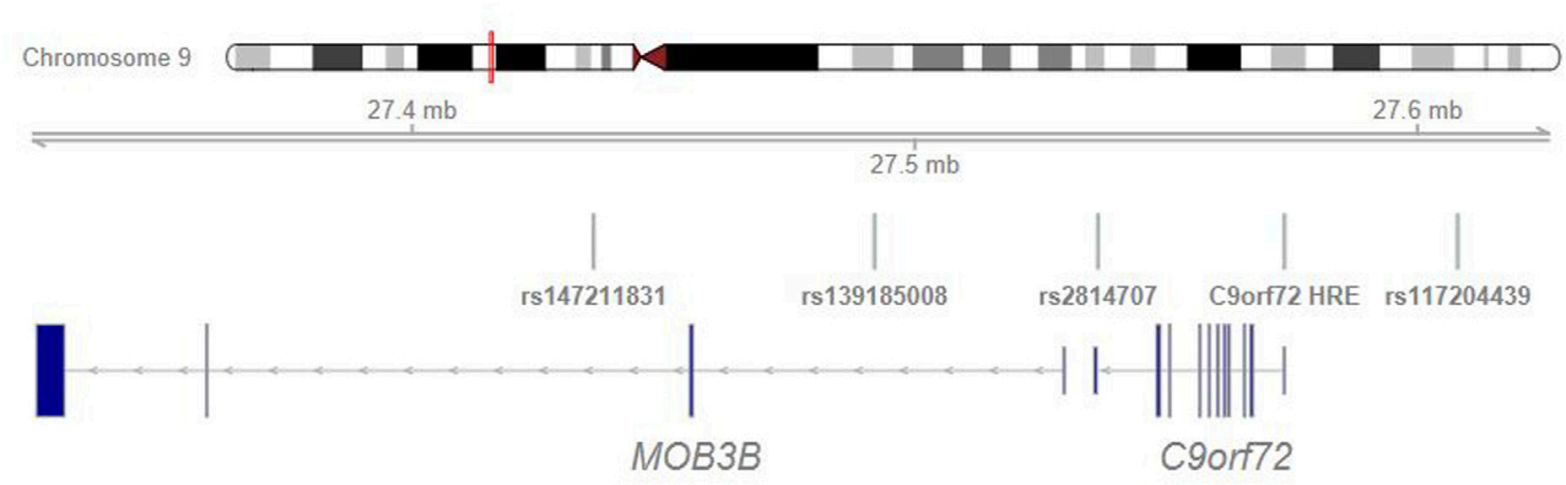
*C9orf72* intermediate allele and expansion tagging SNP positions.

In a previous case-control study from the Netherlands and United Kingdom, rs147211831 and rs117204439 associated with FTD, *C9orf72* HRE and a subset of longer intermediate-length alleles with a median of 12 repeats (Reus et al., 2021). The location of these variants in relation to the HRE and other analyzed variants is shown in Figure 2.

The association of these markers with ALS in our Finnish data was much weaker than the association with rs139185008. The OR conferred by rs147211831 was 2.76 (95% CI 1.54–4.97, *p* = 6.86 × 10^−4^) and by rs117204439 2.05 (95% CI 1.24–3.38, *p* = 5.05 × 10^−3^).

In our Finnish data, these SNPs showed only weak association with the HRE and intermediate-length alleles. Neither SNP tagged consistently longer intermediate alleles, as shown in Figure 1C for rs117204439 which had a stronger association with intermediate-length alleles and had higher MAF among the HRE carriers.

### Finnish biobank data: Association of *C9orf72* HRE and intermediate-length allele tagging SNPs with age

The discovery cohort included 232,878 unrelated Finnish ancestry individuals without diagnosis of ALS or FTD. Rs139185008*C tags the *C9orf72* HRE and longer intermediate-length alleles, and rs2814707*T tags the HRE and intermediate-length alleles with ≥8 repeats.

As shown in Table 1 the frequency of rs139185008*C heterozygotes decreased significantly with age. The difference was statistically significant between the oldest and youngest group (*p* = 0.0001) as well as between the oldest and middle age group (*p* = 0.0005). Rs139185008*C homozygosity was too rare (6-37 individuals per group) for meaningful statistical comparisons.

**TABLE 1:** *C9orf72* intermediate allele tagging rs2814707 and intermediate allele and expansion tagging rs139185008 frequencies in age groups.

				*Oldest* vs*. middle age group*		*Oldest* vs*. youngest age group*	
Discovery cohort	<50 years (n = 64,393)	50–80 years (n = 139,620)	>80 years (n = 28,865)	p	OR [95% CI]	p	OR [95% CI]
rs139185008 heterozygotes	2,129 (3.3%)	4,499 (3.2%)	817 (2.8%)	0.00047	0.87 [0.81–0.94]	0.00011	0.85 [0.78–0.93]
rs2814707 heterozygotes	18,388 (28.5%)	39,311 (28.2%)	8,015 (27.8%)	0.16	0.98 [0.95–1.01]	0.014	0.96 [0.93–0.99]
rs2814707 homozygotes	1,881 (2.9%)	4,296 (2.9%)	858 (3.0%)	0.73	1.01 [0.94–1.09]	0.67	1.02 [0.94–1.11]
Replication cohort	(n = 25,500)	(n = , 46,748)	(n = 8,794)				
rs139185008 heterozygotes	799 (3.1%)	1,473 (3.2%)	240 (2.7%)	0.037	0.86 [0.75–0.99]	0.061	0.88 [0.75–1.01]

Rs2814707 heterozygote frequency decreased also with age and the difference between oldest and youngest group was nominally significant (*p* = 0.014) but did not survive Bonferroni correction (Table 1). When we excluded rs139185008*C carriers from rs2814707*T carriers, the frequencies were 26.7%, 26.3% and 26.2% in individuals age <50, 50-80 and >80 years, respectively (*p* = 0.11, OR = 0.97, 95% CI 0.94–1.01). This finding indicates that the modest age-effect was driven by haplotypes containing rs139185008*C.

**FIGURE 3:**
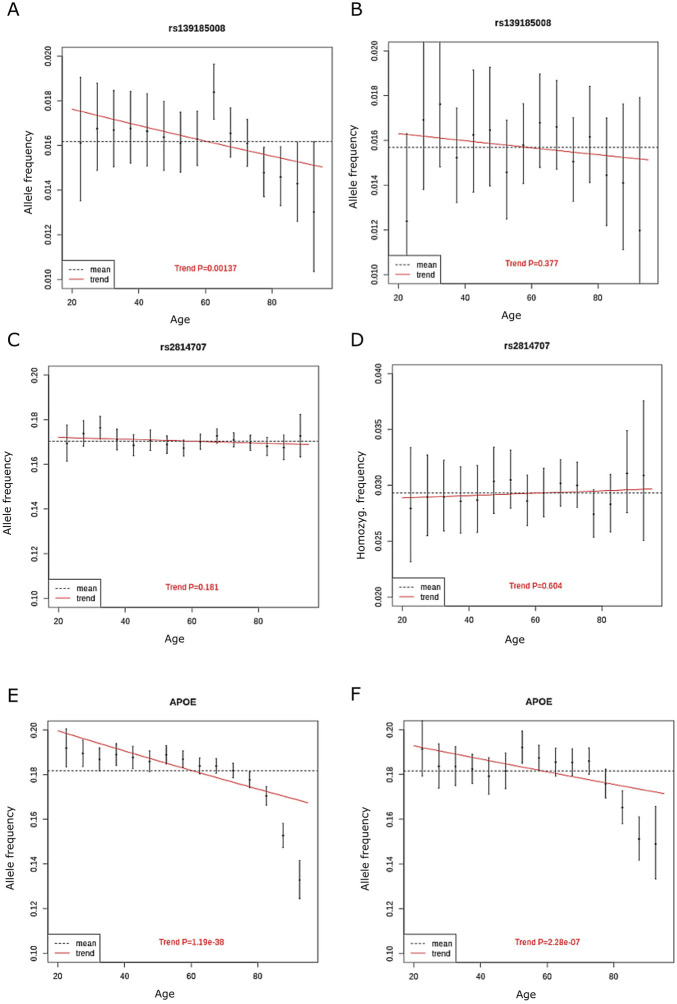
The allele frequency of **(A)** rs139185008*C in discovery cohort in 5-year bins **(B)**, rs139185008*C in replication cohort in 5-year bins **(C)** rs2814707*T n discovery cohort **(D)** rs2814707*T homozygote frequency in discovery cohort **(E)** *APOE ε4* allele frequency in discovery cohort and **(F)** *APOE ε4* allele frequency in replication cohort. The red line shows the trend in allele frequency with age, the allele frequency estimate across all age groups is shown by the dashed line.

We also analyzed allele frequencies across ages 20–95 years in 5-year bins, age groups <20 years and >95 years were excluded due to small number of subjects. The discovery cohort included 230,006 unrelated Finnish ancestry individuals aged between 20 and 95 years and without diagnosis of ALS or FTD. We found that rs139185008 allele frequency decreased significantly by age (*p* = 0.0014, beta = −0.22, standard error = 0.067). In contrast, we rs2814707 homozygosity frequency did not decrease with age (*p* = 0.83) (Figure 3). To compare our findings to a genetic variant with known association with neurodegenerative diseases and aging, we made similar analysis with the frequencies of APOE ε4 allele, which showed a highly significant decrease with age (*p* = 3 × 10^−43^).

### Replication cohort

We set to replicate the decrease in rs139185008*C heterozygote frequency in aging using 80,012 non-overlapping individuals from FinnGen. The rs139185008*C heterozygote frequency decreased with age and the difference was statistically significant between the oldest and middle age group (2-sided *p* = 0.037) but, likely due to smaller number of individuals, only borderline significant in oldest vs. youngest age group (2-sided *p* = 0.061) (Table 1).

We also made analyses on allele frequencies across ages 20–95 years in 5-year bins in the replication cohort. Again, the rs139185008*C heterozygote frequency decreased with age (Figure 3), showed overlapping effect size with the results in discovery cohort but did not reach statistical significance (*p* = 0.38, beta = −0.10, standard error = 0.12). APOE ε4 allele frequencies still associated significantly with age in the replication cohort (*p* = 2.28 × 10^−7^) (Figure 3).

## Discussion

In this study, we first analyzed the best tagging SNPs for *C9orf72* hexanucleotide repeat intermediate-length alleles and the HRE in a Finnish case-control study. Then, we analyzed the effect of these SNPs on longevity in the FinnGen biobank data.

Our results shed light on the haplotype structure of the *C9orf72* HRE and the intermediate-length alleles. We confirmed in an independent dataset that rs139185008 is the best *C9orf72* HRE tagging SNP in the Finnish population. In addition, we observed that the rs139185008 tags longer intermediate-length alleles, especially those with ≥20 repeats. Thus, the longer intermediate repeat alleles and the HRE seem to share a relatively rare haplotype in Finland with a carrier frequency of ca. 3% in controls [HRE carrier frequency estimated ca. 0.2% (Kaivola et al., 2019)]. This observation raises the question, whether the longer intermediate-length alleles (≥20 repeats), which are more common in Finland than in other populations studied (Kaivola et al., 2019), are instable and generate expansions to offspring or mosaic expansions in carriers by somatic instability. It has been shown in transfected cells with varying *C9orf72* hexanucleotide repeat lengths (11, 20, 22 and 41 repeats) that repeat instability increases with longer *C9orf72* repeats and, interestingly, replication fork stalling is observed when there are ≥20 repeats (Thys and Wang, 2015). Rescue of stalled DNA replication is one proposed mechanism for repeat expansions (Mirkin and Mirkin, 2007). Mosaic expansions arising from normal size alleles have been tested in ALS patients spinal cord sections at an estimated detection level of ≥5% mosaicism (Ross et al., 2019). No mosaic expansions were detected in that study, but none of these patients carried intermediate-length alleles of ≥20 repeats (the longest allele was 11 repeats, personal communication by Jay Ross and Guy Rouleau). Testing gonadal and somatic mosaicism in carriers of ≥20 repeat alleles are interesting avenues for future research.

Rs139185008*C was less sensitive marker of the HRE (tagged 80% of HRE) than rs2814707*T (tagged 100% of HRE). As rs2814707 is located closer to the HRE (Figure 2), historical recombination events have most likely occurred between the HRE and rs139185008. The HRE-containing haplotypes seem to differ among European populations. It was previously shown that rs139185008 was not among the top SNPs associated with ALS in the UK Biobank (Rostalski et al., 2021). Here, we tested SNPs identified as HRE-tagging SNPs in a cohort from the Netherlands and United Kingdom. SNPs rs147211831 and rs117204439 tagged the HRE and intermediate-length alleles with a median of 12 repeats and associated with FTD (Reus et al., 2021). In our Finnish data set, these two SNPs showed only weak association with the expansion and did not consistently tag longer intermediate-length alleles (Figure 1C). These two SNPs are located at a longer distance from the HRE than our tagging SNPs and encompass almost 200 kb of DNA (Figure 2), it seems that the extended haplotype structures differ within Europe. However, the core haplotype (<50 kb) has not yet been studied with high resolution, this is becoming possible using e.g. long-read sequencing technologies (Ebbert et al., 2018).

We have previously reported that carrying two copies of the intermediate-length alleles is a risk factor for ALS in Finland, especially when one of the alleles is ≥ 17 repeats (Kaivola et al., 2020). Here, we analyzed this phenomenon using tagging SNPs after exclusion of individuals with the HRE. We found that homozygosity for rs2814707*T was a modest risk factor for ALS (OR 1.84, *p* = 0.012), the carriership of rs139185008*C increased the risk among those homozygous for rs2814707*T (OR = 5.06, *p* = 0.0002). The majority of these subjects had the intermediate-length allele genotype ≥8/≥20 (Figure 1). However, when carriers of rs139185008*C were removed from this analysis the risk conferred by rs2814707*T homozygosity was lost (OR 1.50, *p* = 0.14). This result can be partially due to limited statistical power but indicates that major part of the ALS risk is dependent on the rs139185008*C haplotype structure, which includes the longer intermediate-length alleles. It is of note that our originally reported threshold (≥17 repeats) may not be accurate, the threshold of ≥20 repeats may be more generalizable (de Boer et al., 2020; Kaivola and Tienari, 2022). The caveat of hidden non-genotyped HREs (Rollinson et al., 2015) may play a role in our finding of rs139185008*C heterozygous association with ALS (OR 2.15) since rs139185008*C heterozygotes included 15 subjects (all ALS cases) without intermediate-length alleles. SNP imputation errors may also contribute to this finding. However, hidden HRE should not have a major influence on the results when the subjects are heterozygous for two intermediate-length alleles. SNP and hexanucleotide repeat allele analyses complement each other and a summary of these results is shown in Supplementary Table S1. SNP-based analysis controls for misinterpreted intermediate-length alleles and intermediate-length allele genotype-based analysis controls for non-genotyped HREs (i.e. monoallelic PCR not plausible since ALS risk was associated with heterozygous intermediate-length allele genotypes). These results indicate that hidden HRE is possible in certain genotypes (especially repeat allele homozygotes), but our observations in both intermediate-length allele heterozygotes and SNPs strengthen the evidence for the concept that residual ALS risk may exist at the C9orf72 locus, independent of the HRE. In the future, studying the *C9orf72* intermediate allele and tagging SNP genotype combinations in Finnish FTD cohort will be important to further replicate our findings and C9orf72 haplotypes should be analyzed more in detail to uncover the putative HRE-independent effect of this ALS/FTD locus.

In the FinnGen discovery cohort, we observed that rs139185008*C allele frequency decreased with age, when ALS and FTD diagnoses were excluded. We observed that rs139185008*C allele frequency decreased with age also in the replication cohort but the association was not statistically significant in all tests, which is probably due to the ca. 3-fold smaller cohort size and reduced statistical power. The direction of effect and effects sizes did not much differ in the discovery and replication cohorts. The decrease in rs139185008 minor allele frequency started to decrease after 70 years in both discovery and replication cohorts (Figure 3). This observation suggests that rs139185008*C haplotype may play a role in survival outside ALS/FTD, possibly by increasing the risk for other neurodegenerative diseases. As the estimated prevalence of the HRE is ca. 0.2% (Kaivola et al., 2019) and magnitude of the decrease by age was 0.4%–0.5% it is possible that age-related disease risk is conferred partially by the HRE and partially by haplotypes containing rs139185008*C and longer intermediate-length alleles. We did not observe a decrease in the frequency of rs2814707*T homozygotes. This lack of association with survival can be due to the fact that the vast majority of rs2814707*T homozygotes have intermediate-length alleles with 7–16 repeats, for which the increase in ALS risk was not statistically significant in our previous study (Kaivola et al., 2020). The rs139185008*C haplotypes contributed to the results since the small (non-significant) effect on survival observed in rs2814707*T homozygotes was lost after exclusion of rs139185008*C carriers. Another possibility for the lack of survival effect is that rs2814707*T homozygosity may be a more specific risk factor for ALS/FTD, not for other age-related (>80 years) diseases.

Our study has limitations. We have studied exclusively Finnish individuals and our results may not be generalizable to other populations, not even to other European populations as the *C9orf72* haplotypes seem to differ to some extent. As previously discussed regarding the Finnish ALS case-control cohort (Kaivola et al., 2020), determining *C9orf72* repeat lengths is not always straightforward and genotyping errors are possible and hidden HRE carriers are possible especially in ALS patients carrying rs139185008*C but no intermediate-length alleles. However, the genotyping of the longer intermediate alleles should be reliable, because we performed over-the-repeat PCR and visualized on gel all samples with ≥20 repeats or an expansion to reduce the possibility of mis-genotyping longer intermediate alleles as expansions and *vice versa*. Furthermore, we observed high concordance with RP-PCR based genotypes and AmplideX C9orf72 determined genotypes (Supplementary Table S3). In the biobank study, disease status was derived from national registries and especially FTD cases could have been misdiagnosed as other dementias or psychiatric conditions. Furthermore, even though rs139185008 and rs2814707 imputation INFO scores were good (>0.90), some degree of contamination with wrong genotypes is probable. This would create noise that would most likely cause regression to the mean and decrease the differences between groups rather than increase. The imputation quality is especially important when analyzing rarer variants or variant combinations since in a small cohort each sample and genotype has more impact on the analysis results than in a big cohort. Small samples sizes of the FinnGen cohort were avoided for that reason.

In conclusion, we observed that rs139185008*C tags *C9orf72* HRE and intermediate-length alleles with ≥20 repeats in Finland. Moreover, rs139185008*C frequency decreased with age in a biobank cohort with ALS and FTD diagnoses excluded, indicating population-wide effects in late-onset neurodegenerative diseases as well. In the future, the rs139185008*C haplotypes and risk haplotypes in other populations should be characterized in detail to assess what part(s) of these haplotypes cause increased disease risk.

## Data Availability

The data presented in this study are deposited in GitHub (https://github.com/kkaivola/C9orf72_repeat_length_genotype_data). Biobank data used in this study is available through FinnGen (https://www.finngen.fi/en).
